# Plasticity and dystonia: a hypothesis shrouded in variability

**DOI:** 10.1007/s00221-020-05773-3

**Published:** 2020-03-23

**Authors:** Anna Sadnicka, Masashi Hamada

**Affiliations:** 1 Motor Control and Movement Disorders Group, St George’s University of London, London, UK; 2 Clinical and Movement Neurosciences, UCL Queen Square Institute of Neurology, London, UK; 3 Department of Neurology, University of Tokyo, Tokyo, 113-8655 Japan

**Keywords:** Dystonia, Plasticity, Neurophysiology, Pathophysiology

## Abstract

Studying plasticity mechanisms with Professor John Rothwell was a shared highlight of our careers. In this article, we discuss non-invasive brain stimulation techniques which aim to induce and quantify plasticity, the mechanisms and nature of their inherent variability and use such observations to review the idea that excessive and abnormal plasticity is a pathophysiological substrate of dystonia. We have tried to define the tone of our review by a couple of Professor John Rothwell’s many inspiring characteristics; his endless curiosity to refine knowledge and disease models by scientific exploration and his wise yet humble readiness to revise scientific doctrines when the evidence is supportive. We conclude that high variability of response to non-invasive brain stimulation plasticity protocols significantly clouds the interpretation of historical findings in dystonia research. There is an opportunity to wipe the slate clean of assumptions and armed with an informative literature in health, re-evaluate whether excessive plasticity has a causal role in the pathophysiology of dystonia.

## Introduction

Dystonia is a movement disorder defined by abnormal movements or postures which are caused by sustained or intermittent muscle contractions (Albanese et al. 2013). This pattern of movement can be a physical manifestation of many distinct underlying pathologies. These range from conditions causing widespread neurodegeneration [e.g. neurodegeneration with brain iron accumulation (Schneider et al. 2012)] discrete structural lesions [typically the putamen and globus pallidus (Bhatia and Marsden 1994) ], genetic disorders where there is no overt degenerative change [e.g. DYT1 dystonia (Ozelius et al. 1989)] and functional neurological disorders (Ganos et al. 2014). Pathophysiological investigation of dystonia has focused on the isolated dystonias in which there are no additional neurological features (such as parkinsonism or cognitive involvement). The natural history of isolated (idiopathic or genetic) dystonia is that of an insidious onset with a stable disease course once the symptoms are fully established (usually months–years) (Balint et al. 2018). Non-motor features are present (Eggink et al. 2019) but the most disabling symptoms have dominion over the control of posture and movement and the repercussions this yields for daily life. A unifying pathophysiology model for dystonia remains elusive. However, over the last 2 decades disease models have been dominated by the idea that plasticity regulation is abnormal with excessive plasticity observed within sensorimotor circuits (Quartarone et al. 2006; Quartarone and Pisani 2011; Quartarone and Ruge 2018).

## Origins of the plasticity hypothesis in dystonia

At the turn of the century, abnormalities in inhibition had been noted at multiple levels of the nervous system (Berardelli et al. 1998). However, these were not considered causal to dystonia pathophysiology as reduced inhibition could be experimentally documented outside the clinically involved territory and was also seen in a variety of other unrelated disorders (Berardelli et al. 1998). The influence of sensory factors was also appreciated, as alleviating maneuvers or sensory tricks are a common clinical feature of dystonia [additional sensory input such as light touch substantially improves dystonic contractions (Patel et al. 2014)]. Correspondingly, experimental paradigms probing sensorimotor integration confirmed the importance of the sensory axis within pathophysiological models for dystonia. For example, in primary motor cortex, hyper-excitatory responses to sensory nerve stimulation had been noted (Abbruzzese et al. 2001).

**Fig. 1:**
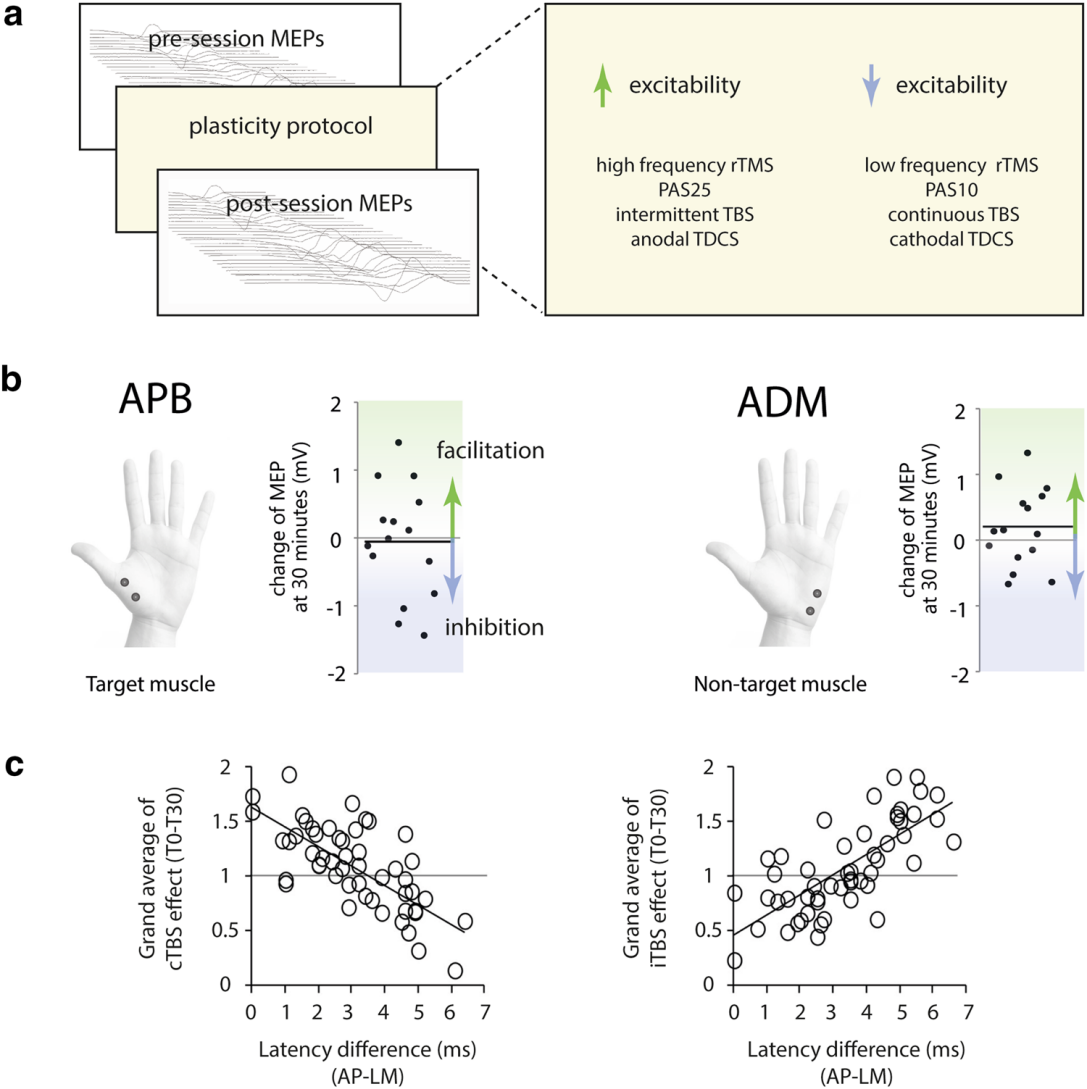
**a** Plasticity paradigms. Examples of non-invasive brain stimulation (NIBS) techniques that induce plasticity. Response is quantified by taking a mean measure (such as amplitude) of the motor evoked potential (MEP) before and after the session. Protocols that are thought to increase excitability include high frequency repetitive transcranial magnetic stimulation (rTMS), paired associative stimulation with an interstimulus interval of 25 ms (PAS25), intermittent theta burst stimulation (iTBS) and anodal transcranial direct current stimulation (TDCS). Protocols that are thought to reduce excitability are low frequency rTMS, PAS with an interstimulus interval of 10 ms, and continuous TBS (cTBS) and cathodal TDCS. **b** Variability of paired associative stimulation (PAS25) in writing dystonia is illustrated in 15 individuals. Each dot represents a single patient’s data and the dark line the group mean. Data for both the target muscle, abductor pollicis brevis (APB) and non-target muscle, abductor digiti minimi (ADM) are shown. **c** Interneuron recruitment and TBS variability. Up to 50% of the variation in TBS was predicted by AP-LM latency, our postulated marker for the efficiency of late I-wave recruitment (see text for detail). Graphs plot the correlation between grand average of cTBS (left) and iTBS effect (right) and AP–LM latency difference

This context therefore provided an exciting milieu when non-invasive brain stimulation (NIBS) techniques were introduced which were thought to examine an analogue of synaptic plasticity at the neuronal level. Repetitive transcranial magnetic stimulation (TMS) and transcranial direct current stimulation (TDCS) techniques appeared to offer the ability to modulate cortical excitability over a time period which outlasted the period brain stimulation itself (Fig. 1a). Changes in excitability were quantified by applying single pulses of TMS to the motor cortex to elicit motor evoked potentials (MEPs) in the muscles of the contralateral hand before and after plasticity protocols (Fig. 1a). At this time quite a few lines of evidence, suggested that these paradigms were a tool by which to probe and modulate synaptic plasticity (Huang et al. 2005; Stefan et al. 2000).

A series of highly influential dystonia publications then ensued. For example low frequency repetitive TMS was tested by Siebner et al. in patients with writing dystonia (Siebner et al. 1999). Rather than the expected decrease in the averaged MEP, patients showed a significant increase in MEPs, suggesting that increased excitability of the motor cortex was important. Quartarone then consolidated this work by applying a paired associative stimulation plasticity (PAS) protocol in writing dystonia in his landmark publication in which they found stronger facilitation of MEP amplitudes in patients compared to controls (Quartarone et al. 2003). This paper cemented excessive plasticity as a leading hypothesis within models for dystonia pathophysiology with the accepting deputy editor of Brain (Professor John Rothwell) commenting that “it was an entirely new idea at the time”. Later publications in writing dystonia consolidated this work suggesting that not only was the magnitude of response excessive but that patients also had abnormal temporal properties and spatial organisation of plasticity responses (Weise et al. 2006). Furthermore, when other dystonia subtypes were tested, such as the cranial and cervical dystonias, these groups were also found to have excessive motor cortex plasticity responses using paradigms which tested the hand muscles (Quartarone et al. 2008). Thus, abnormal excitability was not confined to clinically affected circuits and excessive plasticity was proposed as an endophenotypic trait for dystonia. Another supportive finding was that effective treatment of cervical dystonia with botulinum toxin injections was mirrored by shifts of excessive plasticity responses towards those of controls at the peak of treatment efficacy (Kojovic et al. 2011).

Therefore, when we (and others) found that plasticity responses of the motor cortex could be reduced by cerebellar TDCS in healthy subjects, exploring whether the excessive plasticity responses described in dystonia could be normalised by cerebellar stimulation was an intriguing hypothesis (Hamada et al. 2012). Interestingly, although cerebellar stimulation continued to demonstrate its ability to have a stabilising effect on neurophysiological plasticity response, the premise of the study was undermined by individual variability of plasticity response in patients with writing dystonia (Sadnicka et al. 2014b). In some patients corticospinal excitability after PAS was facilitated (i.e., long-term potentiation-like response), and in some patients it was inhibited (i.e., long-term depression-like response). In fact, no net plasticity response was seen at the group level (Fig. 1b). Our data therefore failed to confirm the expected excessive plasticity in dystonia and revealed marked variability of response instead. Our findings strongly resonated with an emerging appreciation of the inherent variability of NIBS plasticity protocols in health.

## Plasticity-induction by non-invasive brain stimulation is characterised by variability

Historically NIBS protocols were thought to modify corticospinal excitability in a predictable and consistent manner (Pellegrini et al. 2018). However, increasingly inter-individual variability was seen. For example, in one study over 50 subjects were studied with the three most commonly used paradigms to facilitate corticospinal excitability; paired associative stimulation with an interstimulus interval of 25 ms (PAS25), intermittent theta burst stimulation (iTBS) and anodal transcranial direct current stimulation (aTDCS) (Lopez-Alonso et al. 2014). Tellingly, there was no significant effect for any of these paradigms on MEP amplitude (or other neurophysiological markers of excitability) across the whole group (Lopez-Alonso et al. 2014). Within this null result, a mathematical cluster analysis revealed a bimodal response pattern but revealed that only 39%, 45% and 43% of subjects responded with a facilitatory response as expected to PAS25, aTDCS and iTBS respectively (Lopez-Alonso et al. 2014).

The stability of plasticity responses at an individual level is also poor. For example, if individuals have their plasticity response tested at two different sessions using PAS25, the magnitude of evoked plasticity responses are entirely unrelated across the two sessions (Fratello et al. 2006) (other plasticity paradigms such as TDCS are more stable within individuals (Lopez-Alonso et al. 2015)).

Around the time we were studying plasticity in dystonia, we started to study the variability of response to plasticity induction by TBS. Our hypothesis was that some of the inter-subject variability in response to each protocol was due to differences in the population of neurones activated by each TMS pulse. In human motor cortex, TMS of motor cortex evokes several volleys of corticospinal activity which correspond to different MEP latencies. The first volley (direct, D-wave) originates from axonal activation of corticospinal neurones and can be preferentially elicited by applying the TMS coil in a lateromedial (LM) direction. Later volleys (indirect, I-waves) result from activation of mono and polysynaptic inputs to cortical spinal neurones. Early I-waves are preferentially elicited by posterior-anterior (PA) coil placement whereas anterior–posterior (AP) induces later I-waves which are variable between individuals. We had preliminary data to suggest that variability in interneuron recruitment (quantified by AP–LM latency difference) was correlated with TBS response (just 10 subjects at that time). When this was mentioned to Professor John Rothwell at a wine reception at Queen square John said: “DO more!” Accordingly, a TBS variability project was born to establish the relationship between interneuron recruitment and TBS plasticity (Fig. 1c). A few months later, John enquired: “How many people have now been tested for TBS variability?” 42 had been tested with the plan to test 10 more. His retort was: “You have to stop at some point and write a paper!” If John had come by several days later, then the number of subjects in the paper would be more than 52 (Hamada et al. 2013)!

This was one of the now many studies which have contributed to more complete models for the manner in which plasticity is induced by non-invasive stimulation protocols and the factors which account for variability across subjects (Cheeran et al. 2008; Hordacre et al. 2017; Huang et al. 2017; Lopez-Alonso et al. 2014; Muller-Dahlhaus et al. 2008; Wiethoff et al. 2014). For example, non-modifiable (age, gender, genetics) and modifiable (intake of medical and non-medical substances, sleep, state of motor system activation) physiological, technical and statistical factors have all been linked to plasticity variability (Guerra et al. 2020a; Ridding and Ziemann 2010). There is broad consensus that variability in NIBS studies is a consistent and significant research issue (Guerra et al. 2020a). Critically, a more detailed understanding of such variability is fundamental in the study of neurological disorders such as dystonia if meaningful clinical translation is to occur (Guerra et al. 2020b, 2020a).

## Integrating the neuroscientific and dystonia literatures

The movement disorders literature has been slow to adopt this informative literature on the variability of response to NIBS plasticity paradigms. Clinical studies are challenging and access to large numbers of patients is limited unless multicentre studies are performed. However, such variability cannot be dismissed as it likely to be greater in clinical groups with additional clinical factors such as duration and severity of disease and number and type of treatments.

If the dystonia literature is carefully appraised, evidence of variability across patients and study findings has been substantial from the start. For example, early studies within the dystonia literature clearly described large excessive effects with plasticity protocols (Quartarone et al. 2003). However, other studies failed to find any group effect of PAS protocols in patients with focal dystonia or no difference between the response of healthy subjects and those with dystonia (Kang et al. 2011; Sadnicka et al. 2014a). Interestingly, if directly compared, the magnitude of excessive plasticity response documented in some studies that did find a significant difference between controls and patients with dystonia was less than excessive plasticity responses quantified in other studies that found no significance between groups (Meunier et al. 2012; Sadnicka et al. 2014a). Such inconsistencies highlight the problem of attempting to define disease related abnormalities in comparison to a control group when variability is high and the numbers sampled are small.

Other papers emphasised that the abnormality in dystonia may be subtler than a simple increase in plasticity and that the spatial specificity of the response was the core abnormality (for example, patients may have a greater spread of the effect to non-target muscles) (Belvisi et al. 2013; Weise et al. 2006). However, in healthy individuals, plasticity is no longer considered to be specific to the target muscle; arguments that dystonia has a greater spread of response must also account for this finding in healthy subjects (Cheeran et al. 2008). Other disturbances have also observed such as abnormalities in metaplasticity (a synaptic or cellular activity that primes the ability to induce subsequent synaptic plasticity) or homeostatic plasticity (range of plasticity mechanisms that stabilise neuronal activity) yet failed to replicate earlier core plasticity findings (Kang et al. 2011; Karabanov et al. 2015a; Quartarone et al. 2005; Sadnicka et al. 2014a).

## Reappraising the role of plasticity in dystonia

The early literature which cemented the idea that plasticity responses were excessive or non-specific in dystonia has evolved into one of increasing complexity. It is likely that significant inter-subject and intra-subject variability of response to NIBS plasticity paradigms exists in dystonia as it does in healthy controls. In the final section we discuss a number of themes that we believe to be relevant for future investigation.

### Evolving and unchecked story

The plasticity literature in dystonia has often evolved in line with new discoveries without clarity on original findings. Both the strength and the consistency of the association between plasticity responses and dystonia can be questioned. It is likely that we have been sampling a highly variable outcome parameters with numbers that are too low to adequately power studies. Recent reviews have described the belief in the plasticity hypothesis in dystonia as ‘canonical’ rather than evidence based (Conte et al. 2019; Latorre et al. 2019). Our collective commitment to plasticity as a hypothesis for dystonia pathophysiology will lead to both implicit and explicit bias (e.g. the manner with which outlying data is treated, how experiments are planned, which datasets are pushed and accepted for publication) with new research continuing to be framed by the plasticity disease model. Overall the literature may point to increased excitability and/or increased variability of response to protocols which probe motor cortex excitability in dystonia. However, whether plasticity has a causal role in the pathophysiology of dystonia is far from established.

### Specificity

Altered plasticity responses are not unique to dystonia. Abnormalities in plasticity responses have been demonstrated in a multitude of unrelated central nervous system disorders (for example: Alzheimer’s disease (Terranova et al. 2013), autism (Jung et al. 2013), migraine (Pierelli et al. 2013)). We also need to define whether we consider plasticity to be a marker of the syndrome dystonia (a common final manifestation of a range of diseases) or essential to disease pathophysiology for aetiologically homogenous groups such as DYT-1 dystonia (caused by a single gene mutation). If direct pathophysiological insight is to be gleaned, neurophysiological abnormalities specific to a disease need to be identified.

### Limitations of techniques

There is broad agreement that most subtypes of isolated dystonia are likely to represent a network disorder (Jinnah et al. 2017; Sadnicka et al. 2018). Most plasticity work stems from averaged motor evoked potentials, a readout from the motor cortex. What does this signify? Such paradigms look in relative isolation at a single node within the sensorimotor cortex, its data presumably reflecting interactions with other nodes within the dystonic network. Our readout parameter, the motor evoked potential, is a noisy parameter which varies across trials and across individuals. Do current techniques offer limited capacity to get insight into the broader dystonic network?

### Mechanistic correlates

Another complex discussion is how plasticity inducing NIBS protocols relate to plasticity at the synaptic level. It cannot be assumed that changes in the motor cortex measured by shifts in mean MEP are a simple analogue of synaptic plasticity at the cellular level (Carson and Kennedy 2013; Karabanov et al. 2015a; b).

### Cause or consequence?

Most simply, dystonia is a hyperkinetic movement disorder in which there is too much movement with abnormal muscle contractions which lead to abnormal postures. The motor cortex as the common final output that controls movement is therefore likely to be comparatively hyperexcitable as too much movement for given context is being produced. Whether the abnormalities in plasticity response reported are therefore a consequence of the abnormal movements rather than a causal aetiological factor is very difficult to resolve. Any criteria for causal inference are poorly satisfied by our current plasticity literature (Fedak et al. 2015).

### Conceptual considerations

Given such uncertainties it is an interesting academic exercise to examine the plasticity hypothesis in dystonia from a purely theoretical perspective. Would the characteristics of plasticity as we currently understand them offer a good explanation for the disease? For example, task-specific dystonia usually manifests with a stereotypic motor impairment with stability over time which preferentially affects an isolated task. Such features would not be clearly predicted by “runaway” plasticity. Furthermore, if excessive plasticity is an important mechanism, why are many patients resistant to a range of treatments? A system with heightened plasticity could be expected to be more responsive to therapeutic inputs. Finally, the significant phenotypical and aetiological diversity of the dystonias is well documented. Excessive plasticity, especially if parameterised by low dimensional metrics such as changes in mean amplitude of MEPs, is unable to account for such heterogeneity and shouldn’t be used as a mechanism which can span clinical phenotype and aetiology unless the delineating features are established.

## Conclusions

Professor John Rothwell has contributed greatly to the study of plasticity in health and disease and this article has reflected on two decades of research hugely sculpted by his influence. We conclude that as a fundamental mechanism within the brain, synaptic plasticity will never be irrelevant to the mechanism, manifestation or treatment of dystonia. However, the early literature which cemented the idea that plasticity is excessive in dystonia has evolved into one of increasing complexity as high variability of response to NIBS techniques significantly clouds the interpretation of findings. Studies in healthy controls increasingly characterise the extent and mechanisms behind inter-subject and intra-subject variability of plasticity response. There is therefore an important opportunity to wipe the slate clean of assumptions in dystonia research and to re-evaluate the validity of the plasticity hypothesis armed with this informative literature in healthy controls.
